# Comparative Evaluation of Multidetector Computed Tomography and Dual-Energy Computed Tomography Findings in Gastrointestinal Tuberculosis

**DOI:** 10.7759/cureus.32149

**Published:** 2022-12-03

**Authors:** Asif Khan, Sachin Khanduri, Surbhi ., Harleen Chawla, Sonal Kaushik, Zaara Khan, Rohit ., Shreya Chitravanshi, Uzma Kabir, Danish Ansari

**Affiliations:** 1 Radiodiagnosis, Era's Lucknow Medical College and Hospital, Lucknow, IND; 2 Radiology, Era's Lucknow Medical College and Hospital, Lucknow, IND; 3 Radiodiagnosis, Era’s Lucknow Medical College, Lucknow, IND

**Keywords:** dual-energy computed tomography (dect) providing high-quality images, sputum acid- fast bacilli (afb) assessment, gastroduodenal tuberculosis, small bowel tuberculosis, peritoneal tuberculosis

## Abstract

Aim: To compare multidetector computed tomography (MDCT) and dual-energy computed tomography (DECT) imaging findings in gastrointestinal (GI) tuberculosis.

Objective: To study imaging findings of MDCT and DECT in GI tuberculosis.

Methodology: All the patients falling in the sampling frame and fulfilling the eligibility criteria were clinically examined and demographic details, presenting complaints, medical history, history of anti-tubercular treatment (ATT) intake, personal habits, and family history of tuberculosis were noted. All the patients underwent sputum acid-fast bacilli (AFB) assessment. Outcomes of investigations like bronchoscopy and fine-needle aspiration cytology (FNAC)/biopsy were also noted wherever available. Ascitic fluid AFB and culture assessments were also performed wherever feasible. All CT scans were performed on a 384-slice dual-energy CT scanner (Somaton Force, Siemens Healthcare) and all the images were post-processed on a workstation using syngo.via software that allows the analysis of images using three material decompositions. Features like peritonitis, lymph node involvement, GI wall thickening, and solid organ involvement were focused on. Subjective assessment of images of both MDCT and DECT were assessed by two experienced radiologists to prepare the CT diagnosis. The mutual agreement of the two observers was considered final.

Conclusions: The findings of the study showed that both MDCT, as well as DECT, were useful in the diagnosis of GI tuberculosis. On the basis of these findings, DECT could be considered to have an edge over MDCT in the diagnosis of GI tuberculosis. Keeping in view the small sample size and high prevalence, further studies on a larger sample size with relaxed sampling criteria are recommended to validate the findings of the present study.

## Introduction

Tuberculosis (TB) has affected humans for most of their history and remains a major cause of mortality in adults worldwide [1,2]. This incremental trend marks the impact of systematic efforts to curb and control the disease. The occurrence of the majority of TB cases in low and middle-income countries has established a relationship between the degree of advancement, basic infrastructure, quality of life in a nation , and efficiency of control measures for TB [3]. Indian scenario concerning tuberculosis is much worse, according to an estimate by whom India represented over 26% of the global TB burden [4]. Moreover, extrapulmonary TB (EPTB) which can be traced back to 1643 when the autopsy on Louis XIII showed ulcerative intestinal lesions associated with a large pulmonary cavity, the ever-growing incidence of the tubercular disease has seen a rise in cases of extrapulmonary TB [5]. According to an estimate, 15% of all tubercular cases develop extrapulmonary tuberculosis, thus making the screening of extrapulmonary TB in tuberculosed cases much more important. Though EPTB can affect a wide variety of organs, the most common site of infection is the abdomen, followed by the pleura and lymph nodes [6]. The incidence of abdominal TB is higher due to easy contraction of the disease by ingestion/digestion of infected sputum or proximity with the infected. The pathogenesis of EPTB is attributed to the bacteria reaching the gastrointestinal (GI) tract via the hematogenous spread, ingestion of infected sputum, or direct spread from infected contiguous lymph nodes and fallopian tubes [7-9]. The most common site of involvement of GI tuberculosis is the ileocecal region [10]. Though CT has emerged as one of the most commonly used techniques to detect GI and abdominal TB, the varied methods of CT itself cause different diagnostic results and hence need to be evaluated for their accuracy. Two such methods are multidetector CT (MDCT) and dual-energy CT (DECT). Though both the techniques are similar at the core, the difference lies in how they collect and process the image, wherein DECT an additional attenuation measurement is obtained at a second energy, allowing the differentiation of the two materials; in MDCT a single tube potential switching is used to allow alternate projection measurements to be acquired at low and high tube potentials, the low and high-energy data sets are acquired simultaneously [11,12].

## Materials and methods

This is a cross-sectional study, carried out on a total of 52 patients falling in the sampling frame and fulfilling the eligibility criteria, for 24 months. The study was carried out at the Department of Radiodiagnosis in collaboration with the Department of Medicine in Era’s Lucknow Medical College & Hospital, Lucknow. Clearance for carrying out the study was obtained from the Institutional Ethical Committee Era's Medical College (Approval number: ELMC & H /RCELL, EC/2021/132), and informed consent was obtained from all the patients. All the patients were clinically examined and demographic details, presenting complaints, medical history, history of anti-tubercular treatment (ATT) intake, personal habits, and family history of tuberculosis were noted. All the patients underwent sputum acid-fast bacilli (AFB) assessment. Outcomes of investigations like bronchoscopy and fine-needle aspiration cytology (FNAC)/biopsy were also noted wherever available.

All CT scans were performed on a 384-slice DECT scanner (Somaton Force, Seimens Healthcare) and all the images were post-processed on a workstation using syngo.via software that allows the analysis of images using three material decompositions. Subjective assessment of images of both MDCT and DECT was assessed by two experienced radiologists to prepare the CT diagnosis (more than five years of experience). The final diagnosis was based on clinical response to ATT treatment and/or a proven culture-positive biopsy. Inclusion and exclusion criteria are mentioned in Table 1.

**Table 1:** Inclusion criteria and exclusion criteria. FNAC: fine-needle aspiration cytology

S. No	Inclusion criteria	Exclusion criteria
1	All patients referred from the medical and surgical dept for suspected abdominal tuberculosis	Pregnancy
2	Demonstration of acid-fast bacilli in ascitic fluid	Allergic to dye
3	Growth of mycobacterium tuberculosis on culture in ascitic fluid	Deranged kidney function test
4	Satisfactory therapeutic response to chemotherapy in patients with clinical/laboratory/radiological evidence of gastrointestinal tuberculosis	Patient with known bowel malignancy
5	FNAC and biopsy-proven gastrointestinal tuberculosis	Critically ill patient

Statistical analysis

**Figure 1:**
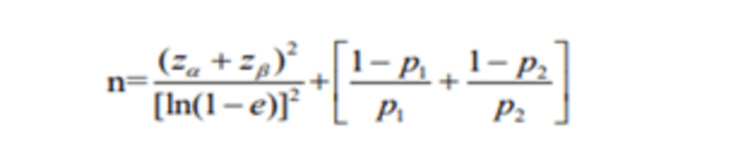
Formula for calculating sample size. Where p1 = 0.571 (57.1%) the proportion of lymphadenopathy in MDCT, p2 = p1 under the null hypothesis, proportion difference e = 60% of p1 considered to be clinically significant, type I error, α=5% (level of significance), type II error β=20% for setting power of study 80%, data loss factor = 10%, the sample size was calculated to be n = 52. MDCT: multidetector CT

The sample size was calculated based on the proportion of Lymphadenopathy in MDCT and considering the null hypothesis of equality between MDCT and DECT findings using the formula (Figure 1).

## Results

The present study was carried out on a total of 52 clinically suspected cases of GI tuberculosis (age range: 19-59 years; 80.8% males and assessed using MDCT and DECT. All the cases also underwent sputum AFB, bronchoscopy, FNAC/biopsy, and ascitic fluid (AFB and culture) assessment for tuberculosis.

On DECT, lymph node involvement (76.9%), GI wall thickening (67.3%), and peritonitis (32.7%) were the major findings. Solid organ involvement was seen in a total of nine (18.1%) cases - maximum (n = 6) had involvement of the liver followed by spleen (n = 2) and both liver and spleen (n = 1), respectively. The large intestine (63.5%) was found to be the most affected GI part followed by the small intestine (15.4%), a combination of the large and small intestine (11.5%), stomach (7.7%), and a combination of large and small intestine with other GI parts (1.9%) respectively. Diagnosis of tuberculosis was made in 35 (67.3%) cases. Out of these, two were diagnosed with pulmonary tuberculosis and 35 were diagnosed with GI tuberculosis. Of the remaining 17 cases, six (11.5%) were diagnosed with colitis, three (5.8%) each was diagnosed with appendicitis and typhoid respectively, two (3.8%) each with gastritis and neoplasm, and one (1.9%) was diagnosed as pancreatitis. MDCT findings suggestive of peritonitis, lymph node involvement, and GI wall thickening were revealed in 20 (38.5%), 44 (84.6%), and 41 (78.8%) patients. A total of 9/52 (17.3%) cases showed solid organ involvement. The liver alone was involved in six (34.6%) cases followed by involvement spleen alone (3.8%) and both the liver and spleen (1.9%), respectively. Large intestine (73.1%) was the most commonly affected site followed by small intestine (7.7%), both large and small intestine (7.7%), stomach (5.8%), and large intestine with colon and other sites in one (1.9%) patient. Diagnosis of tuberculosis was made in 39 (75%) cases. Out of these, two were diagnosed with pulmonary tuberculosis and 37 were diagnosed with GI tuberculosis. In the remaining 15 cases, four (7.7%) were identified as colitis, three (5.8%) as neoplasia, and two (3.8%) each as enteritis and gastritis. There was one case each diagnosed as appendicitis and pancreatitis.

**Figure 2:**
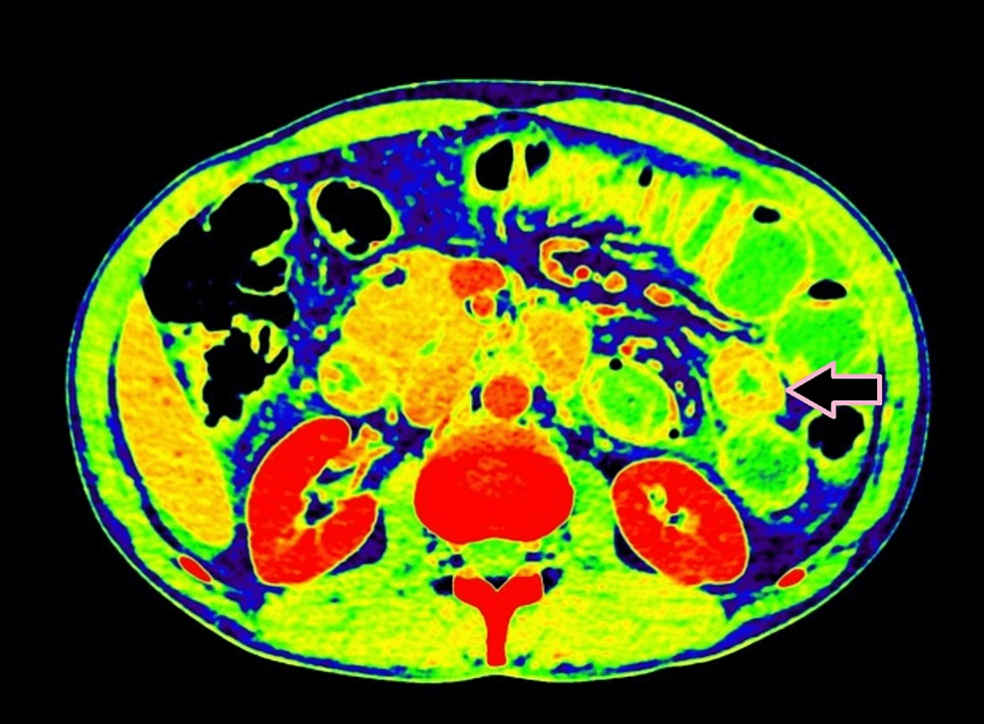
Axial DECT image on the basic atomic number of different metabolites present within the cells showing circumferential mural thickening of jejunum with luminal narrowing (case 1). DECT: dual-energy computed tomography

**Figure 3:**
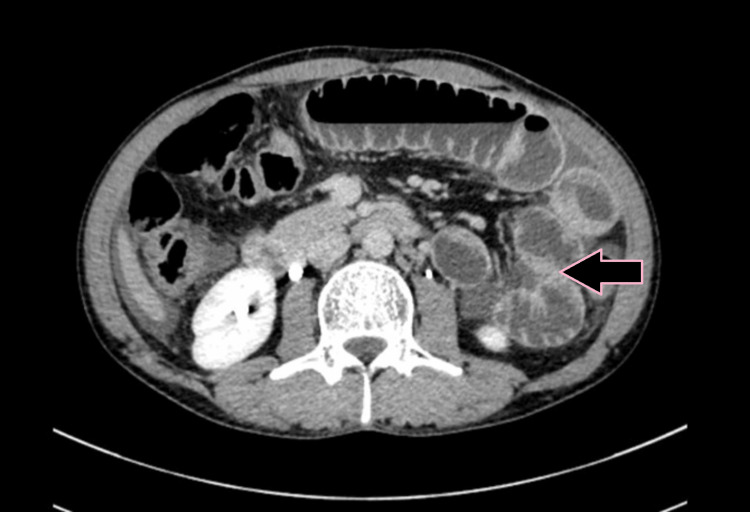
Axial DECT image showing circumferential mural thickening of jejunum with luminal narrowing (case 1). DECT: dual-energy computed tomography

**Figure 4:**
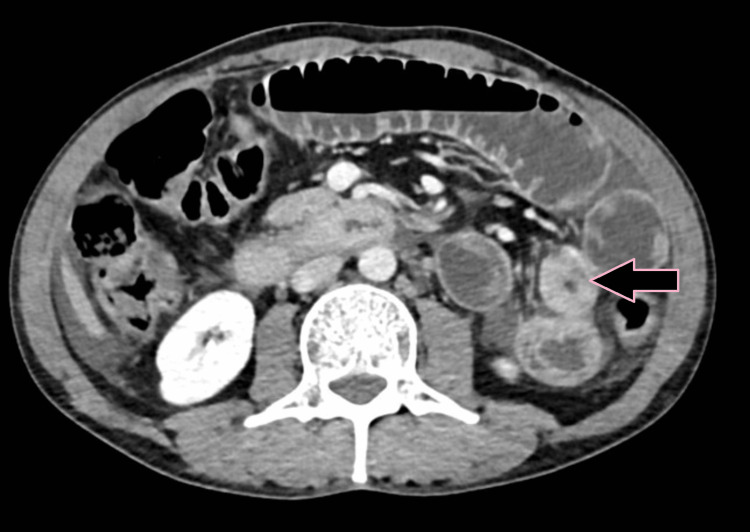
Axial CECT image showing circumferential bowel wall thickening of the jejunum (pink arrow) with a normal adjacent small bowel loop indicated for comparison (case 1). CECT: contrast-enhanced computed tomography

**Figure 5:**
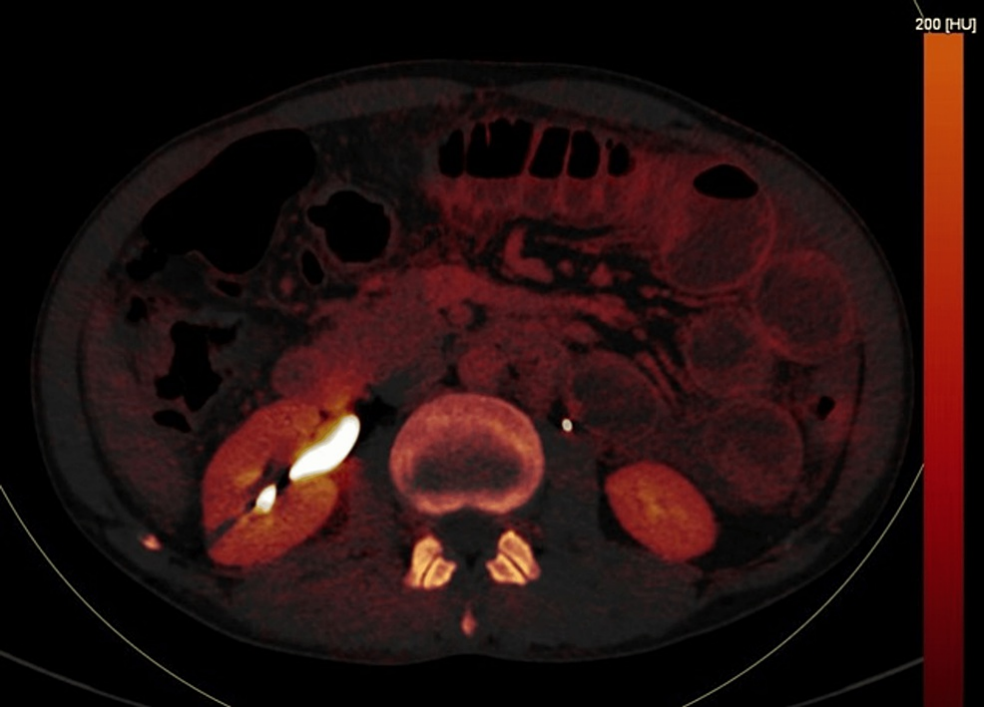
Axial iodine overlay image showing visible iodine color in the circumferential thickened wall of the bowel compared to the color evident in the adjacent uninvolved bowel, indicating differential enhancement due to bowel thickening (case 1).

**Figure 6:**
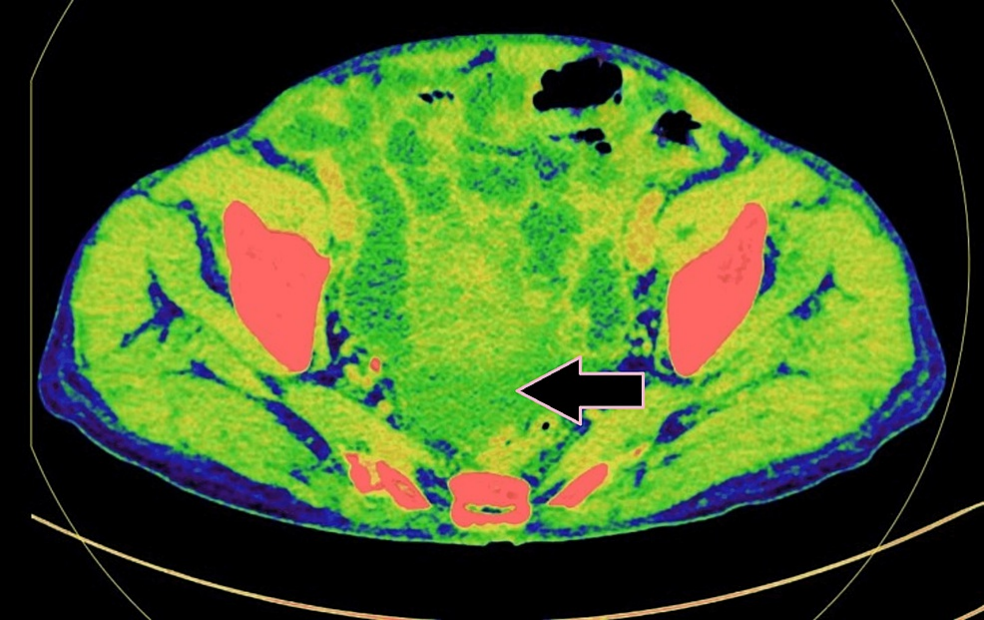
Axial color code image based on the atomic number shows ascites (case 2).

**Figure 7:**
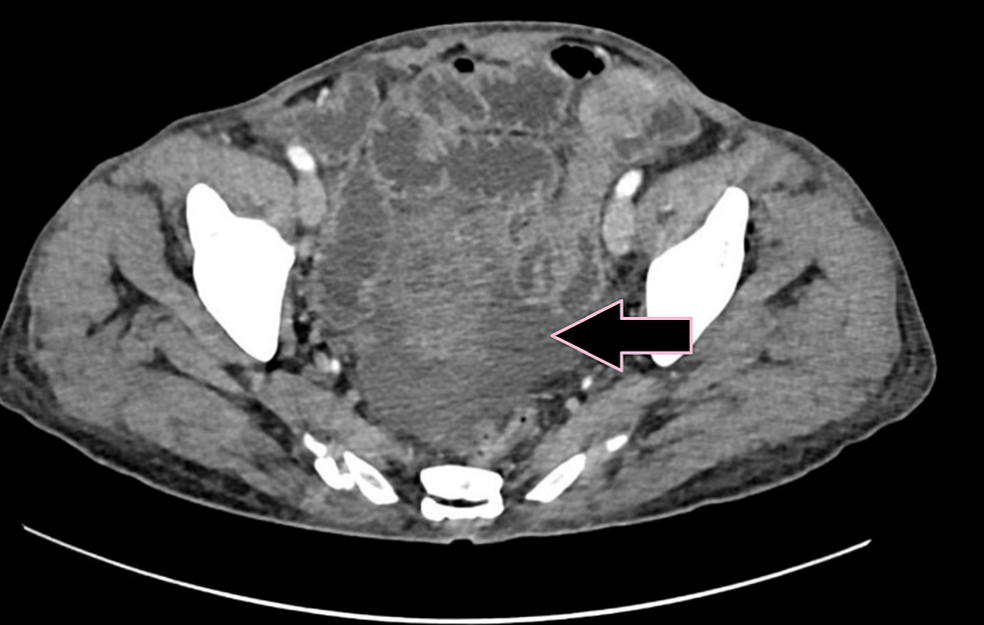
Axial DECT image showing free fluid in peritoneal cavity—ascites (pink arrow) (case 2). DECT: dual-energy computed tomography

**Figure 8:**
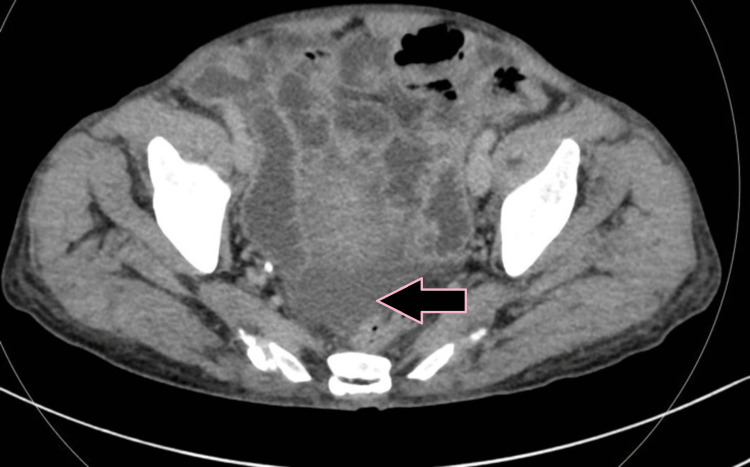
Axial MDCT with contrast showing ascites in the peritoneal cavity (case 2). MDCT: multidetector computed tomography

**Figure 9:**
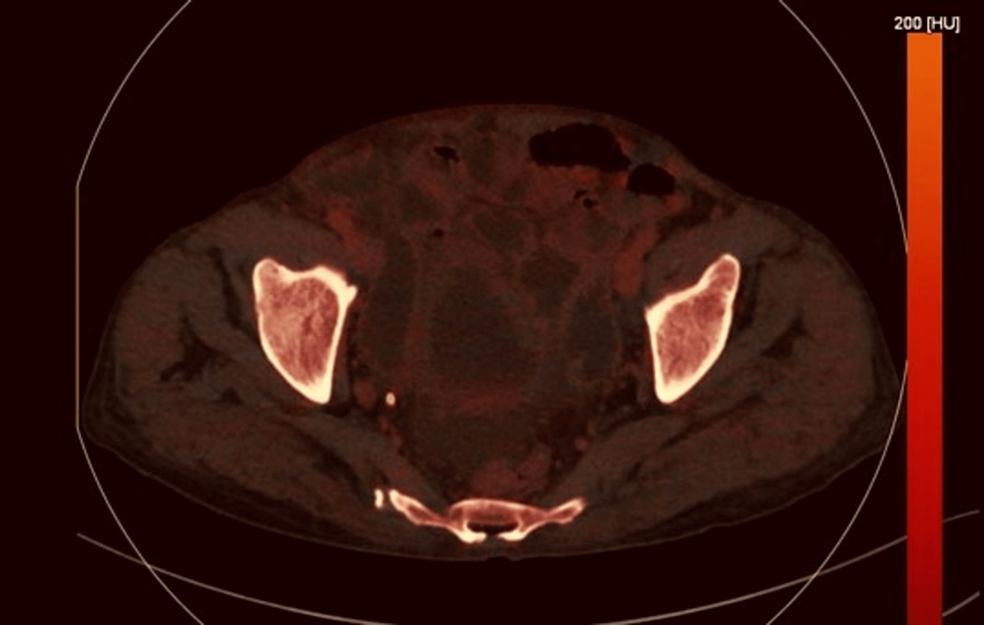
Axial iodine overlay image show ascites (case 2).

**Figure 10:**
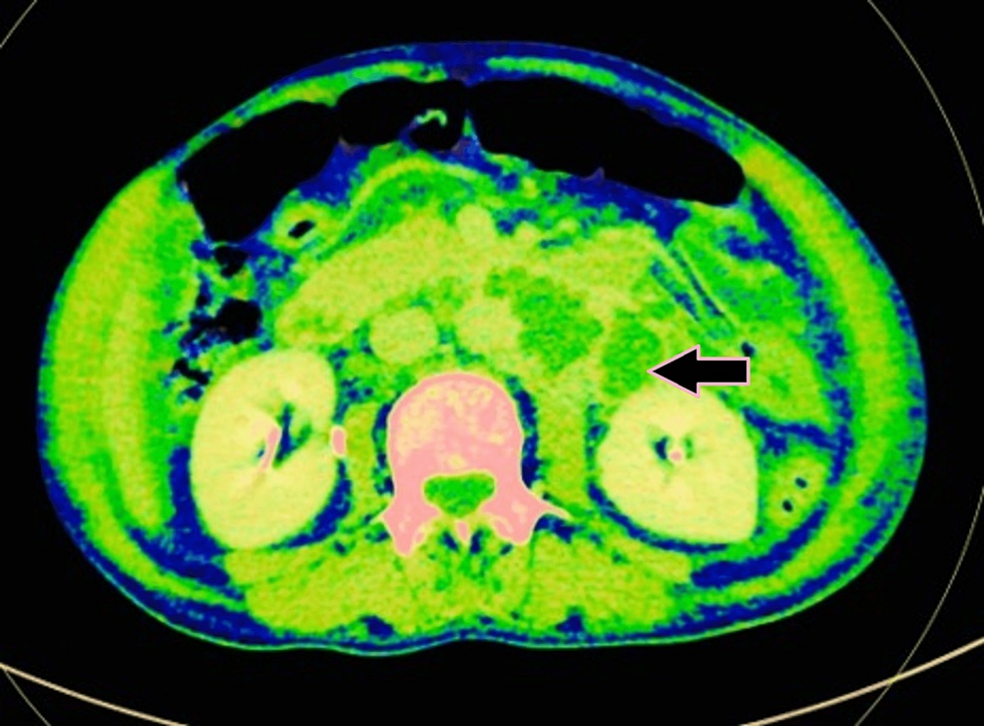
Axial color code image based on the atomic number shows lymphadenopathy from abdominal tuberculosis (case 3).

**Figure 11:**
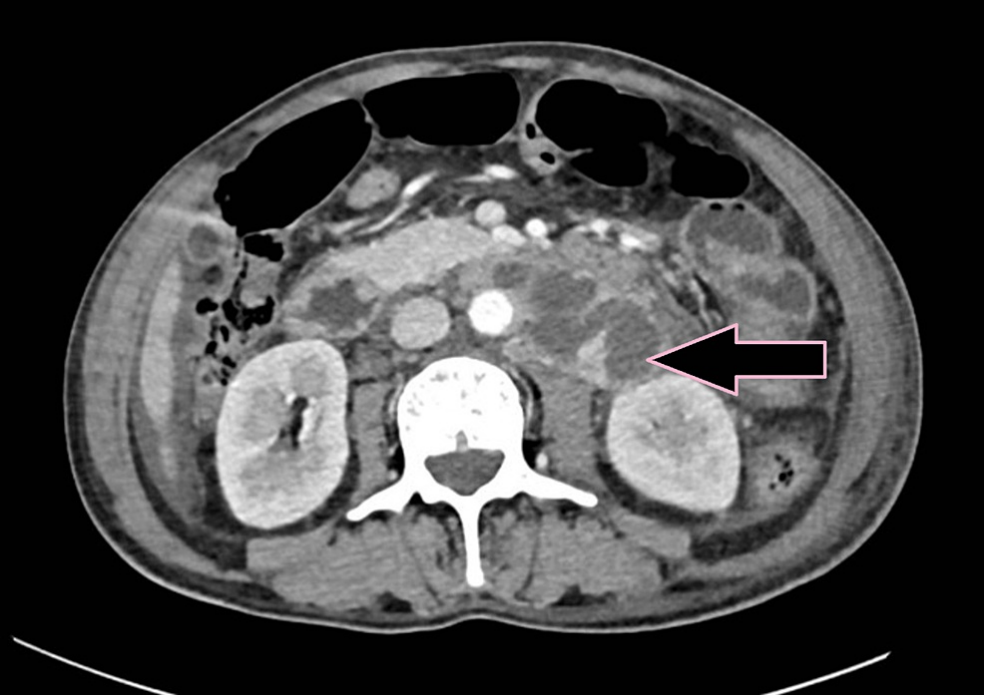
MDCT: contrast-enhanced CT scan shows a lymph node with peripheral enhancement and a low-attenuation center (case 3). MDCT: multidetector computed tomography

**Figure 12:**
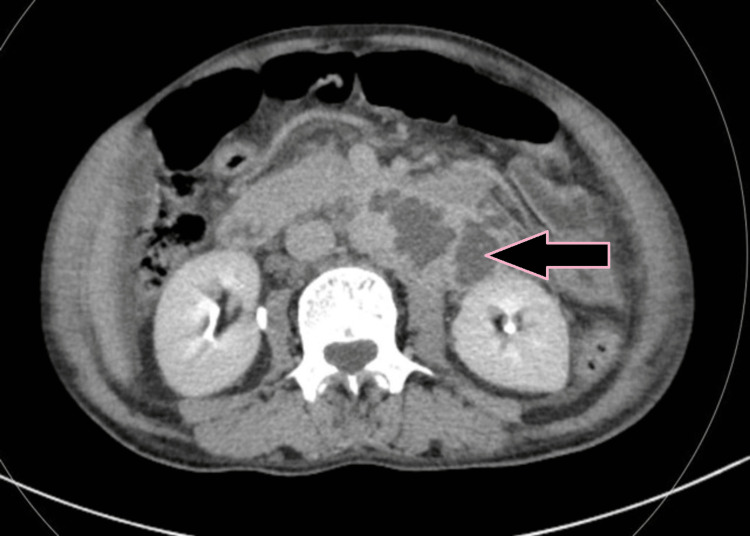
DECT shows lymph nodes with characteristic low attention center and peripheral rim (case 3). DECT: dual-energy computed tomography

**Figure 13:**
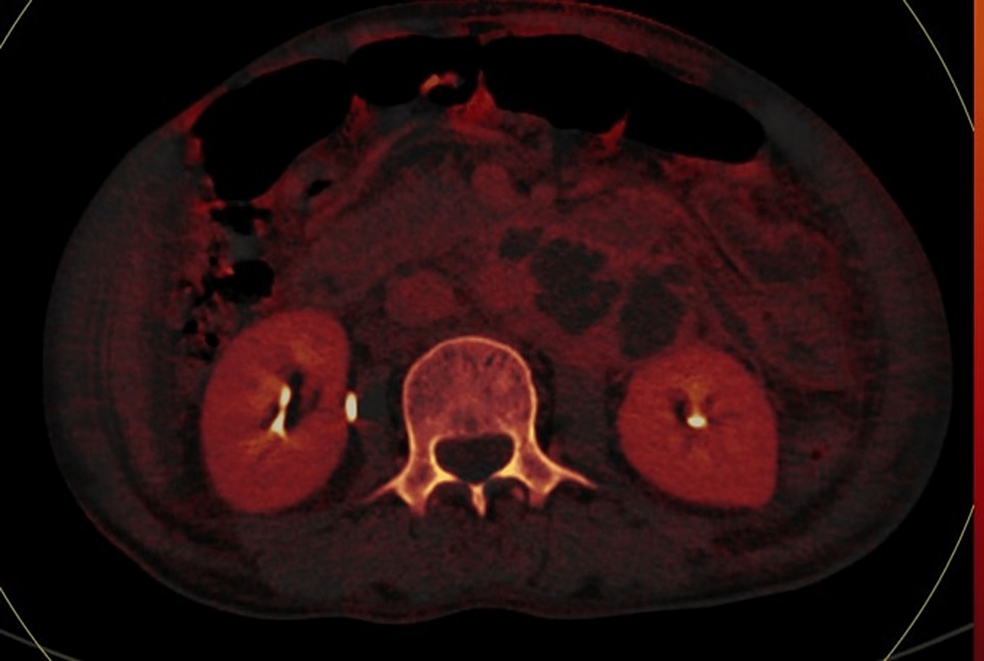
Axial iodine overlay image shows visible iodine color in lymphadenopathy from abdominal tuberculosis (case 3).

As compared to a confirmed diagnosis, MDCT had 30 true positives, seven false positives, two false negatives, and 13 true negative cases of GI tuberculosis. Correspondingly, MDCT was 93.8% sensitive and only 65% specific in the diagnosis of GI tuberculosis. The positive and negative predictive values were 81.1% and 86.7%, respectively. The diagnostic accuracy of MDCT was calculated as 82.7%. There was a strong agreement between MDCT and the final diagnosis. As compared to a confirmed diagnosis, DECT had 30 true positives, three false positives, two false negatives, and 17 true negative cases of GI tuberculosis. Correspondingly, DECT was 93.8% sensitive and only 85% specific in the diagnosis of GI tuberculosis. The positive and negative predictive values were 90.9% and 89.9%, respectively. The diagnostic accuracy of DECT was calculated as 90.4%. There was a strong agreement between DECT and the final diagnosis Tables 2-3). The accuracy of MDCT for the diagnosis of GI tuberculosis was 82.7% [5]. As compared to the final diagnosis, DECT was 93.8% sensitive and 85% specific for the diagnosis of GI tuberculosis. It had positive and negative predictive values of 90.9% and 89.5%, respectively. The accuracy of DECT for diagnosis of GI tuberculosis was 90.4%. As compared to MDCT diagnosis, DECT was 71.2% sensitive and 86.7% specific for the diagnosis of GI tuberculosis. It had positive and negative predictive values of 93.9% and 68.4%, respectively. The accuracy of DECT for diagnosis of MDCT-diagnosed GI tuberculosis was 84.6% (Table 4). In one patient, circumferential mural thickening of the jejunum with luminal narrowing was detected (Figures 2-5). In the other patient, tubercular ascitic fluid was detected (Figures 6-9) and in another patient was detected by a lymph node with peripheral enhancement and a low-attenuation center (Figures 10-13).

**Table 2:** Comparison of MDCT diagnosis of GI tuberculosis against confirmed GI tuberculosis diagnosis. GI: gastrointestinal; MDCT: multidetector computed tomography; Sens: sensitivity; Spec: specificity; PPV: positive predictive value; NPV: negative predictive value.

MDCT Diagnosis	Confirmed Diagnosis		Total	
	GI Tuberculosis	No Tuberculosis		
GI Tuberculosis	30	7	37	
No Tuberculosis	2	13	15	
	32	20	52	
Sens	Spec	PPV	NPV	Accuracy
93.8	65	81.1	86.7	82.7

**Table 3:** Comparison of DECT diagnosis of gastrointestinal tuberculosis against confirmed gastrointestinal tuberculosis diagnosis. GI: gastrointestinal; DECT: dual-energy computed tomography; Sens: sensitivity; Spec: specificity; PPV: positive predictive value; NPV: negative predictive value.

DECT Diagnosis	Confirmed Diagnosis		Total	
	GI Tuberculosis	No Tuberculosis		
GI Tuberculosis	30	3	33	
No Tuberculosis	2	17	19	
	32	20	52	
Sens	Spec	PPV	NPV	Accuracy
93.8	85.0	90.0	89.5	90.4

**Table 4:** Comparison of MDCT and DECT Findings for diagnosis of gastrointestinal tuberculosis. GI: gastrointestinal; MDCT: multidetector computed tomography; DECT: dual-energy computed tomography; Sens: sensitivity; Spec: specificity; PPV: positive predictive value; NPV: negative predictive value.

DECT Diagnosis	MDCT Diagnosis		Total	
	GI Tuberculosis	No Tuberculosis		
GI Tuberculosis	31	2	33	
No Tuberculosis	6	13	21	
	37	12	52	
Sens	Spec	PPV	NPV	Accuracy
71.2	86.7	93.9	68.4	84.6

## Discussion

Interestingly, although tuberculosis affects both pulmonary as well as extrapulmonary regions, it is pulmonary tuberculosis that remains the mainstay of the diagnostic and controlling maneuvers and hence extrapulmonary tuberculosis remains a less commonly attended part of tuberculosis diagnostic strategies. Radiologic imaging is invaluable for the diagnosis of GI tuberculosis. Abdominal ultrasound and CT are the most used techniques. On a CT, abdominal TB is often characterized by peritoneal disease, free or loculated ascites, and high attenuation fluid because of its large protein and cellular content. Peritoneal nodular thickening and enhancement are other common findings seen through CT [13]. Lymphadenopathy is the most common radiologic finding visualized by CT in abdominal tuberculosis [14]. In the present study, we found that DECT was more specific in the detection of different abnormal conditions, and hence the number of cases diagnosed as GI tuberculosis was fewer in DECT (n = 33) as compared to MDCT (n = 37). The confirmed diagnosis based on clinical and laboratory assessment was made in 32 cases. One of the reasons for the DECT confirmed as compared to MDCT confirmed cases was that DECT was able to provide the reconstructed images that were able to provide high-quality results without the use of contrast. DECT provides high-quality images even after eliminating the need for oral contrast, does not require multiple exposure phases, and highlights the presence of pathologies more precisely [15]. Early diagnosis and adherence to treatment regimens are of key importance for a quick and efficient recovery, however, with varying presentations, it is common for EPTB to be confused with other conditions such as inflammatory bowel disease, cancer, and other infectious diseases [16]. The abdominal presentation may involve different structures such as the GI tract, genitourinary tract, solid organs (liver, spleen, pancreas), gallbladder, aorta and its branches, peritoneum, and lymph nodes, frequently with concomitant involvement of those organs. The disease may mimic several other conditions such as lymphoma, Crohn’s disease, amoebiasis, and adenocarcinoma, among others. Imaging findings are not pathognomonic but may be highly suggestive of the disease as considered in conjunction with clinical findings, immunological conditions, and the demographic origin of the patient [17].

Owing to this feature, DECT was able to distinguish between artifacts and actual abnormalities. Moreover, it also provided a better image quality that was helpful in qualitative assessment too. As far as the detection of a higher number of lymph nodes, peritoneal abnormality, and wall thickness on MDCT is concerned, it was because of the limitation of MDCT to provide better quality images even with the use of contrast whereas DECT was able to distinguish between the artifacts and actual pathologies even without the use of contrast.

Limitation of the study: The sample size was very small and the unable to take a proper follow-up CT scan of the patient after having treatment. Though various diagnostic techniques have been evaluated by contemporary studies, very few studies have compared the two different CT techniques. Moreover, as there is immense pressure for quick and timely diagnosis of tuberculosis and EPTB, it is the need of the hour to identify the technique which can provide timely diagnosis at efficient cost and the least number of visits. The present study is being carried out with the aim to compare the findings of MDCT and DECT in GI TB.

## Conclusions

In the present study, we observed that both MDCT, as well as DECT, achieved the same sensitivity (93.8%) however, DECT emerged as more specific (85%) than MDCT (65%). These outcomes were based on a mix of objective as well as qualitative assessments of images. Through this study, we also endorse the value of qualitative assessment rather than focusing on the objective features alone and recommend they be interpreted in holistic terms. The findings of the study showed that both MDCT, as well as DECT, were useful in the diagnosis of GI tuberculosis. On the basis of these findings, DECT could be considered to have an edge over MDCT in the diagnosis of GI tuberculosis. Keeping in view the small sample size and high prevalence, further studies on a larger sample size with relaxed sampling criteria are recommended to validate the findings of the present study.

## References

[1] Holloway KL, Henneberg RJ, de Barros Lopes M, Henneberg M (2011). Evolution of human tuberculosis: a systematic review and meta-analysis of paleopathological evidence. Homo.

[2] Comas I, Coscolla M, Luo T (2013). Out-of-Africa migration and Neolithic expansion of Mycobacterium tuberculosis with modern humans. Nat Genet.

[3] Rathi P, Gambhir P (2016). Abdominal tuberculosis. J Assoc Physicians India.

[4] Rajendran H, Razek AAK, Abubacker S (2019). Multimodal imaging of fibrosing mesenteric tuberculosis. Radiol Case Rep.

[5] Sunkara A, Wagh DD, Singhal S (2009). Abdominal tuberculosis: a panoramic view. Int J Surg.

[6] Chong VH, Lim KS (2009). Gastrointestinal tuberculosis. Singapore Med J.

[7] Sheer TA, Coyle WJ (2003). Gastrointestinal tuberculosis. Curr Gastroenterol Rep.

[8] Sharma SK, Mohan A (2004). Extrapulmonary tuberculosis. Ind J Med Res.

[9] Chugh SN, Jain V (2007). Abdominal Tuberculosis — Current Concepts in Diagnosis and Management. Medicine Update.

[10] Dhar P (1998). Abdominal tuberculosis. Ind J Tub.

[11] McCollough CH, Leng S, Yu L, Fletcher JG (2015). Dual- and multi-energy CT: principles, technical approaches, and clinical applications. Radiology.

[12] Carmi R, Naveh G, Altman A (2005). Material separation with dual-layer CT. IEEE Nucl Sci Symp Conf Rec.

[13] Akhan O, Pringot J (2002). Imaging of abdominal tuberculosis. Eur Radiol.

[14] Joshi AR, Basantani AS, Patel TC (2014). Role of CT and MRI in abdominal tuberculosis. Curr Radiol Rep.

[15] Baş S, Zarbaliyev E (2021). The role of dual-energy computed tomography in locating gastrointestinal tract perforations. Cureus.

[16] da Rocha EL, Pedrassa BC, Bormann RL, Kierszenbaum ML, Torres LR, D'Ippolito G (2015). Abdominal tuberculosis: a radiological review with emphasis on computed tomography and magnetic resonance imaging findings. Radiol Bras.

[17] Gulati MS, Sarma D, Paul SB (1999). CT appearances in abdominal tuberculosis: a pictorial essay. Clin Imaging.

